# How Does B Cell Antigen Presentation Affect Memory CD4 T Cell Differentiation and Longevity?

**DOI:** 10.3389/fimmu.2021.677036

**Published:** 2021-06-10

**Authors:** Robin A. Welsh, Nianbin Song, Scheherazade Sadegh-Nasseri

**Affiliations:** ^1^ Graduate Program in Immunology, Johns Hopkins School of Medicine, Baltimore, MD, United States; ^2^ Department of Biology, Krieger School of Arts & Sciences, Johns Hopkins University, Baltimore, MD, United States; ^3^ Department of Pathology, Johns Hopkins School of Medicine, Baltimore, MD, United States

**Keywords:** memory, CD4 lymphocyte, gene regulation, longevity, B cell Ag presentation, new CD4 memory markers, resting memory CD4+ T-cells

## Abstract

Dendritic cells are the antigen presenting cells that process antigens effectively and prime the immune system, a characteristic that have gained them the spotlights in recent years. B cell antigen presentation, although less prominent, deserves equal attention. B cells select antigen experienced CD4 T cells to become memory and initiate an orchestrated genetic program that maintains memory CD4 T cells for life of the individual. Over years of research, we have demonstrated that low levels of antigens captured by B cells during the resolution of an infection render antigen experienced CD4 T cells into a quiescent/resting state. Our studies suggest that in the absence of antigen, the resting state associated with low-energy utilization and proliferation can help memory CD4 T cells to survive nearly throughout the lifetime of mice. In this review we would discuss the primary findings from our lab as well as others that highlight our understanding of B cell antigen presentation and the contributions of the MHC Class II accessory molecules to this outcome. We propose that the quiescence induced by the low levels of antigen presentation might be a mechanism necessary to regulate long-term survival of CD4 memory T cells and to prevent cross-reactivity to autoantigens, hence autoimmunity.

## Dendritic Cells as Antigen Presenting Cells to Initiate a Primary Response

Initiation of an adaptive immune response begins with naïve T cells being activated by antigens presented on dendritic cells (DCs), a highly specialized professional antigen presenting cell (APC) (1, 2). As a frontline defender, DCs are key APCs bridging the gap between innate and adaptive immunity. Located primarily in peripheral tissues, immature DCs are well known for their ability to recognize and capture invading pathogens mainly through phagocytosis and micropinocytosis. Uptake of antigen (Ag) is closely followed by upregulation of MHC Class I, Class II and co-stimulatory molecules on the surface of DCs, as they lose the ability to perform macropinocytosis (3, 4). Mature DCs then migrate to draining lymph nodes where they present pathogen-derived epitopes to naïve CD4 and CD8 T cells (5). One unique characteristic of DCs is their ability to uptake Ag *via* phagocytosis and cross-present it on MHC Class I molecules. This makes DCs a perfect primary antigen presenter for initiation of an immune response (2). Moreover, it has also been noted that activation of immature DCs by various Toll-like receptor ligands (TLR3 and TLR9) transiently increases antigen specific micropinocytosis (6), which likely increases the ability of DCs to capture Ag within an inflammatory context. To date, research into what contributions DCs make to memory development has been limited and mainly focuses on memory CD8 T cells development (7–11). Use of Batf3 knock-out (KO) mice, which lack CD8a DCs responsible for cross-presentation, found no impact on primary CD4 T cell responses but drastically impaired CD8 responses (12). Likewise, work using *Toxoplasma gondii* showed a crucial role for CD4 T cells in protecting Batf3 KO mice from succumbing to *T. gondii* infection (13). Yet none of these studies using DC KO mice investigated a role for DCs in memory CD4 T cell development. Data from Dalai et al., however suggests that loss of DCs does not likely impact the formation of memory CD4 T cells as removal of CD11c+ DCs did not affect the development of quiescent memory CD4 T cells (14).

## B Cells as APCs in Secondary Responses

B cells are another major professional APCs, which unlike DCs, take up antigens specifically by B cell receptor (BCR) (1). Upon interaction with a cognate Ag, the BCR-Ag complex would be internalized and shuttled to the specialized MHC class II enriched compartments (MIIC) for processing and presentation to the Ag-specific CD4 T cells (15). These CD4 T-B interactions provide essential activation signals to B cells for affinity maturation and differentiation into memory B, or antibody-secreting plasma B cells (16). The memory B cells generated from this T-B interaction have been found to also be important for CD4 T cell memory responses (17).

## How B Cells and DCs Impact Memory T Cell Development

It is generally accepted that memory T cells differentiate after exposure to Ag followed by multiple rounds of proliferation (18–20). While characterization of memory T cells has been explored intensely, the onset of differentiation of Ag-experienced T cells into memory, and how APCs influence this process is less appreciated. Especially that in rare publications, it has been proposed that CD8 memory T cells may be generated upon asymmetric cell division, which precludes the need for interaction with antigen presenting cells (14, 21, 22). On another line of studies, CD8 memory T cell development and homeostasis has been reported to be mediated by IL-15Rα expressed by DCs and Macrophages (23, 24). It is also found that long-lasting CD8 memory can be achieved in the absence of CD4 T cells or B cells (25).

For CD4 Memory T development, however, TCR-pMHC interaction appears to drive CD4 Memory T development (14, 26–30). In this regard, Williams et al. found that lower levels of LCMV antigen density led to high functional avidity CD4 T memory differentiation, while higher levels of LCMV antigen density promoted both high avidity and low avidity CD4 T cells expansion (28). However, the authors did not explore whether DCs or B cells were the APCs to drive such differentiation. Studies addressing contributions of B cells to activation of naïve CD4 T cells has been inconclusive (31). Conversely, several investigations have reported that B cells play a critical role in regulating CD4 memory T development and differentiation (14, 17, 26, 30, 32–37). It is noteworthy that among these studies, both Chowdhury (17) and Misumi (35) found that absence of antigen specific B cells either from SCID mice without B cells or treatment of anti-CD20 mAb did not impact the priming of CD4 T cells in viral infection but impaired the development and effector function of memory CD4 T cells. By virtue of having antigen specific B Cell Receptors, B cells can recognize and internalize specific antigens, process, and present them to cognate CD4 T cells (15). As such, B cell antigen presentation adds a new and exciting dimension to our current knowledge.

The first clear demonstration that B cells play a role in memory CD4 T cell generation/differentiation came from Bradley and colleagues who reported B cell knockout mice did not develop memory CD4 T cells (32). Further studies have shown that loss of B cells adversely affects development of Tuberculosis (TB)-specific CD4 memory precursor effector cells (MPECs) in TB vaccinated B cell deficient mice (36). Because of the ability of B cells to produce antibodies that bind to Ag, it has been postulated that contribution of B cells to CD4 memory T cell development might be linked to Ag-Ab complexes. However, when this issue was specifically addressed by Whitmire et al., T cell responses to lymphocytic choriomeningitis virus (LCMV) infection, the team found that in contrast to B cell-deficient mice, membrane Ig expressing Tg mice retained functional Th cell memory, indicating that B cells selectively preserve CD4 T cell memory independently of immune complex formation (33).

To directly test if B cells were important for the development of CD4 T cell memory, Dalai et al. tested the specific interactions of various APCs with Ag experienced CD4 T cells (14). Using an *ex vivo* anergy assay, the group showed that only B cells, but not DCs, induced a resting state in Ag experienced CD4 T cells. Further *in vivo* characterization using an adoptive cell transfer assay further confirmed the *ex vivo* observations. Previous findings had demonstrated that sub-optimal levels of agonist peptides had induced a resting state in T cells *in vitro*, and *in vivo* (34, 38–44). Thus, the above observations that B cells, but not DCs, pulsed with low doses of Ag induced resting memory CD4 T cells confirmed prior findings that B cells are indispensable for memory CD4 T cell development/differentiation. In agreement with the above findings, B cell deficient mice did not develop quiescent CD4 memory T cells. However, when B cells were transferred to the B cell deficient mice, hyporesponsive CD4 memory T cells were developed. Importantly, B2 (B220+CD43+) follicular B cells, which have diverse BCR were identified as the cells that rendered CD4 memory T cells hyporesponsive (14). These finding were later supported by Keck et al, who found that B cells were required for both optimal expansion and T-bet expression in response to weak TCR stimulation and optimal generation of CD4 T memory (30).

## Contribution of Ag Density Presented by Follicular B Cells to CD4 Memory T Cell Induction/Differentiation

Building upon those initial findings, Dalai et al. tested the effects of B cell presentation of peptide-MHC (pMHC) density on the induction of quiescent memory CD4 T cells. They used a clever strategy by recovering B cells from mice at various timepoints post immunization and transferring them into recipient mice harboring CD4 T memory precursor cells at 4-day intervals (26). This staggered timeframe allowed Dalai et al. to correlate the amount of pMHC presented by the B cells to the time post immunization; earlier time points displayed more pMHC, and later time points fewer pMHC. Interestingly, the group found that only B cells harvested between day 16-20 post OVA immunization induced resting hyporesponsive CD4 memory T cells. These findings supported the idea that CD4 memory T cells are signaled to a resting state by the presentation of a subthreshold numbers of pMHC. These conclusions were further expanded to HEL-specific B cells (45) HEL-specific B cells when used for induction of quiescence/resting state of Ag experienced T cells were more efficient in capturing the Ag and induced quiescence in Ag experienced CD4 T cells at much later time points, i.e., 41-48 days *vs* 16-20 days post immunization by non-specific B cells. In those experiments B cells immunized with protein antigens were transferred to mice that carried primed T cells at 4-day intervals. The rationale was to find out when during an immune response B cell presentation of pMHC reaches to the levels necessary for the induction of quiescence naturally, *in vivo*. It was quite gratifying to see that HEL-specific B cells had captured far more antigen so that the required densities of pMHC for inducing quiescence had reached 20 plus days later than the polyclonal B cells (26). Altogether, Dalai et al. established that: (1) B cells are the APCs responsible for rendering CD4 memory T cells the quiescent, and (2) low levels of pMHC presentation are the main driving force that signal CD4 T cells to enter a resting state (26).

**Figure 1:**
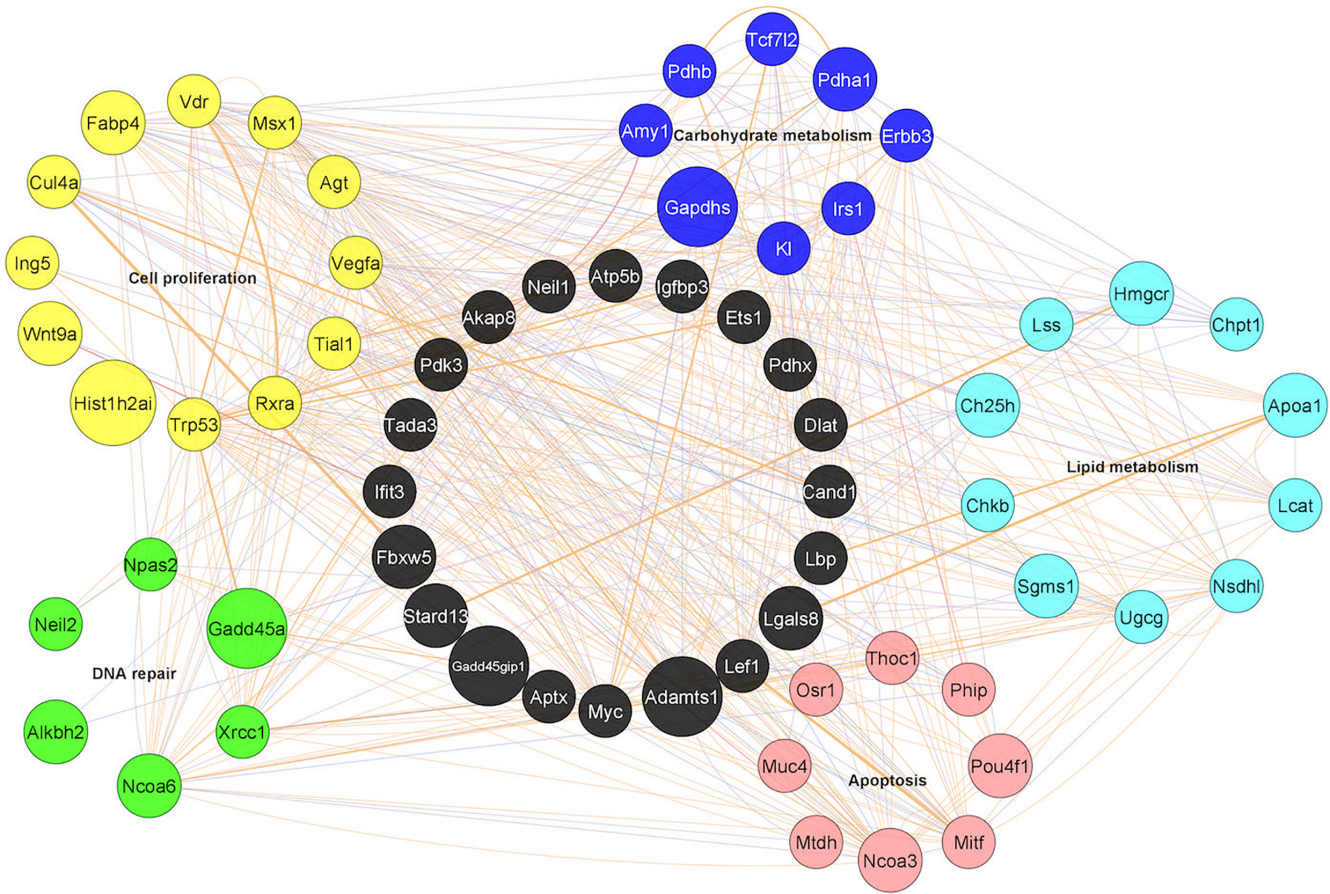
Gene networks in long-lived CD4 memory T cells. Five different gene programs were identified as dynamically regulated during memory CD4 T cell differentiation. The genes shown were from the long-lived memory CD4 cells 10.5 months post immunization as compared to naïve controls and are marked in different colors: Yellow: cell proliferation; Green: DNA repair; Red: Apoptosis; Light blue: Lipid metabolism; Dark blue: Carbohydrate metabolism; Black: Not identified in the five gene programs but served as connecting genes. Each line represents an interaction/co-expression of genes as identified by literature report.

More recently, we have explored how this state of anergy impacts both the longevity and function of CD4 T memory cells. Song et al. investigated gene expression dynamics in CD4 T memory cells at different stages post immunization representing activated, early memory, late memory, and long-term memory stages (46). OVA-specific DO11.10 T cells were adoptively transferred into naïve mice before infecting them with Vaccinia-OVA virus, followed by harvesting the CD44^hi^DO11.1^pos^ T cells at different time points post immunization and subjecting their mRNA for gene expression analyses. Through this approach, the group was able to illustrate the gene expression dynamics occurring during CD4 T memory development up to almost 1 year. In agreement with findings of others (47–51), authors found that the OVA-specific CD4 memory T cells adopted a resting phenotype. Furthermore, the memory phenotype associated with multiple genetic programs regulating cellular proliferation, DNA repair, prevention of apoptosis, glucose, and lipid metabolism (**Figure 1**). Specifically, most genes regulating cellular proliferation and DNA repair response were found to be associated with p53 pathways, which highlights the importance of limiting cell proliferation and promoting DNA repair in long-lived CD4 Memory T cells. Also, of note was that like CD8 Memory T cells, genes regulating lipid metabolism were upregulated indicating that long-lived CD4 Memory T cells may also rely on lipid metabolism. However, unlike CD8 memory, the genes regulating lipid metabolism in CD4 T memory were found to be centered on regulating cellular cholesterol and ceramide levels, which could be related to the T cell signaling and prevention of apoptosis. Altogether, these programs play important roles in CD4 Memory T development and maintenance.

The above genetic studies also revealed upregulated levels of CD99, CCR10 and Itga3 as potentially new surface markers for long-lived CD4 memory T cells. Importantly, the high expression levels of these new CD4 memory markers at the protein level were confirmed to hold true across different animal models and antigens. For example, CD99hi resting human CD4 T cells from flu vaccinated donors had much better proliferation responses than the CD99lo CD4 subsets to *in vitro* challenges, indicating that the gene expression programs found in murine CD4 memory T cells could also be applicable to human CD4 memory T cells. Overall speaking, this work indicated not only that the resting state of CD4 memory T cells was mediated by multiple genes and could be part of the reason for CD4 memory longevity, but also the surprising findings that the murine CD4 memory differentiation is regulated by genetic programs that evolve upwards of 6 months to fully appear.

## Contribution of Class II Accessory Molecules in CD4 Memory Formation

The finding that proper development of CD4 T memory cells relies on quantitative differences in presentation of immunodominant epitopes by B cells, brings the focus to the potential roles that accessory molecules in antigen processing play in the selection of epitopes for binding to MHC Class II. It is demonstrated that as the main Class II peptide-editor, HLA-DM (human DM; murine H2-M) contributes to the selection of immunodominant epitopes by generating higher quantities of those epitopes (52–55). HLA-DO (human DO; murine H2-O), is a second accessory molecule, which requires DM for its expression; DO is mainly expressed in thymic epithelium and B cells (54–56). Both DM and DO contribute to T cell immunity in a significant way, because lymphocytes usually respond to a small portion of the potential determinants on a protein antigen, defined as ‘*immunodominant*’ (57). Immunodominant epitopes are the essential targets of the immune response against infectious diseases, cancer, autoimmune diseases, and allergy. Hence, deserve the attention devoted to the understanding of epitope selection and immunodominance. To better understand how each accessory molecule impacts immunodominant epitope selection, we must discuss each molecule individually.

### Mechanism of DM in Finding the Immunodominant Determinants During Antigen Processing

It has been well established that the MHC II groove is flexible and requires a bound peptide to maintain its shape. Without a peptide, the MHC II groove would close and becomes inefficient in binding peptides (58–60). Thus, newly synthesized MHC II molecules bind to a domain of the Class II invariant chain (CLIP) that serves two functions; a) protects the groove from binding to peptide in the ER (61), and b) acts as a place-keeper, while another domain of Ii guides the complex to the specialized vesicular compartments filled with pathogen-derived antigenic peptides, MIIC. Within MIIC, DM is necessary to first dissociate CLIP to form a peptide-receptive conformation that can quickly scan unfolded exogenous proteins to find its suitable determinant (62). DM does this job by effectively dissociating any peptide sequences that do not fill in the pockets of the MHC II groove. Only when a sequence of antigenic determinant that would fit in the MHC II groove leading to formation of a compact folded conformation, the complex becomes resistant to DM-mediated dissociation (*DM-resistant*). Next, the proteases would trim the MHC II bound determinant. The proteases also cut the antigenic determinants that do not fit the groove, hence are susceptible to DM-mediated dissociation (*DM-sensitive*) and are dislodged by DM (63–71). The solution of the crystal structure of the DM/DR complex (72) using DR1/peptide complexes that enforced an open DR1 groove, revealed that DM would bind the P1 pocket of HLA-DR molecules tightly if empty, and would remain bound until a P1 filling peptide would bind the groove and induce closing of the groove, and displacing DM (72–74). The above findings were complemented by the measured thermodynamics of peptide binding to DR1, indicating that a greater entropic penalty, versus a smaller penalty, was associated with structural rigidity rather than with the flexibility of the pMHC complexes (75). These findings suggested that an overall dynamic MHC II conformation in addition to P1 pocket occupancy, determines susceptibility to DM-mediated peptide exchange and provides a molecular mechanism for DM to efficiently target poorly fitting pMHC II complexes and editing them for more stable ones. Hence, in addition to the removal of CLIP, DM helps in shaping epitope selection and immunodominance by producing a higher abundance of those determinants (62).

### Different Models on How DO Fine-Tunes Antigenic Epitope Selection

DO also contributes to the selection of immunodominant epitopes, although understanding the contributions of DO to epitope selection has proven to be highly challenging (54–56, 76). In brief, our knowledge about DO can be distilled into two working hypotheses: (1) DO binds to DM to inhibit its activity, mainly removal of the CLIP peptide and, (2) DO differentially affects presentation of structurally diverse peptides and acts as a second accessory molecule working together with DM in fine tuning MHC II repertoire selection. Data in support of the former hypothesis mainly comes from studying over-expression of DO genes in cell lines, or dendritic cells (77, 78); Welsh, 2019 #13} and the recent mutagenesis and structural studies of DM/DO interactions (79, 80). The 3D structure of DM/DO showed that DO binds to DM at the same interface with which DM interacts with DR1 (74). Studies supporting the latter hypothesis came from biochemical (81) and biophysical studies demonstrating that DO only affected *association* kinetics of certain peptides to DR but, had no effect on the *dissociation* kinetics of any tested peptide/DR1 complexes (76, 82). The effects of DO on association kinetics directly correlated with peptide sensitivity to *DM-mediated dissociation*. DO reduced binding of peptides that formed *DM-sensitive* complexes with DR and enhanced the binding of peptides that formed *DM-resistant* complexes. In a nutshell, it was clearly shown that; i) DO works directly on DR1, and not by regulating the effect of DM, ii) DO can only bind the *peptide-receptive* MHC Class II, and iii) that this *peptide-receptive* conformation is generated by DM. Hence, authors proposed that DM and DO cooperate for a more effective epitope selection. Thus, in one model, DO would reduce presentation of *immunodominant epitopes*, whereas in the other, DO would increase the abundance of immunodominant epitopes.

### Speculations for Future Research

The question of the potential contributions of DO and DM to memory CD4 T cell development is of most interest and is discussed below. A few characteristics of DO hint to its possible link to CD4 memory differentiation. First, DO is mainly expressed in B cells (81, 83, 84) and it enhances the presentation of immunodominant epitopes (56, 76). Next, it has been documented that successful entry of B cells into the germinal center (GC) requires high expression levels of pMHC (85–89). B cells enter GC and interact with CD4 T cells in search of proper signaling for affinity maturation. It is conceivable that CD4 T cells also receive signals from GC B cells for their own differentiation into resting memory T cells. One might say if high levels of pMHC equip B cells for entry into GC, how could B cells signal T cells to differentiate into resting memory, as this process requires suboptimal densities of pMHC presentation. An answer worth considering is that once B cells enter GC, their expression levels of DO and DM decreases, leading to a reduced level of pMHC II expression (90–92). As such, those GC B cells can interact with Ag-specific CD4 T cells in the Light Zone (LZ), selecting them to become memory precursor cells. In support of this argument, in an elegant study, Kim et al. have documented that memory CD4 T cells bear high affinity TCR for pMHC II (27), hence memory CD4 T cells are selected based on TCR affinity. One may predict that alterations in this controlled entry into the GC reaction could lead to faulty CD4 T cell memory development and possibly the development of increased autoreactivity.

Since biology tends to repeat itself, it would be interesting to compare the effects of pMHC numbers on APCs and their effects on CD8 memory T cell development. While as far as we know no studies has made such data available, in an exciting new study authors reported that in the absence of B cells CD8 T Cell memory formation was compromised, while CD8 effector function was enhanced. One might speculate that since CD4 T cells are essential for CD8 memory T cell development (93), perhaps their contributions to CD8 memory is mediated indirectly *via* CD4 memory T cells.

Future experimental evidence is needed to clarify the proposed relationship of these MHC II accessory molecules to the development and maintenance of CD4 memory T cells, and hopefully this review would prompt new research on the qualitative and quantitative antigen presentation on CD8 memory T cell development.

## Author Contributions

All authors listed have made a substantial, direct, and intellectual contribution to the work and approved it for publication.

## Funding

Supported by grants from NIAID, R01AI063764, R21AI101987, and R01AI120634, to SS-N.

## Conflict of Interest

The authors declare that the research was conducted in the absence of any commercial or financial relationships that could be construed as a potential conflict of interest.
